# Decreasing but still significant facilitation effect of cold-season macrophytes on wetlands purification function during cold winter

**DOI:** 10.1038/srep27011

**Published:** 2016-06-01

**Authors:** Xiangxu Zou, Hui Zhang, Jie Zuo, Penghe Wang, Dehua Zhao, Shuqing An

**Affiliations:** 1 School of Life Sciences, Nanjing University, Nanjing 210046, PR China

## Abstract

To identify the facilitation effect of a cool-season aquatic macrophyte (FE_am_) for use in effluent purification via constructed floating wetlands (CFWs) and to determine the possible pathways used during a winter period with an average temperature of less than 5 °C, pilot-scale CFWs were planted with the cold-season macrophyte *Oenanthe clecumbens* and were operated as batch systems. Although some leaves withered, the roots retained relatively high levels of activity during the winter, which had average air and water temperatures of 3.63 and 5.04 °C, respectively. The N and P removal efficiencies in CFWs decreased significantly in winter relative to those in late autumn. The presence of cool-season plants resulted in significant improvements in N and P removal, with a FE_am_ of 15.23–25.86% in winter. Microbial N removal accounted for 71.57% of the total N removed in winter, and the decrease in plant uptake was the dominant factor in the wintertime decrease in N removal relative to that in late autumn. These results demonstrate the importance of cold-season plants in CFWs for the treatment of secondary effluent during cold winters.

Due to the high concentrations of pollutants, such as total nitrogen (TN), metals and disinfection byproducts^1^,^2^,^3^, effluent from secondary wastewater treatment plants (WWTPs) does not reach the standard of wastewater reclamation for direct and indirect reuses, which represent one of the alternatives for mitigating water shortage problems in China. However, direct loading of secondary effluent into natural watercourses or reservoirs can put great stress on the natural ecosystems, and WWTPs have become a source of pollution instead of purification with respect to protecting aquatic ecosystems from eutrophication^4^. Therefore, conducting further treatment of secondary effluent before loading into natural systems is critical. Due to the characteristics of secondary effluent, such as high TN and low biological oxygen demand (BOD), constructed wetlands have been recognized as more cost-effective and efficient than the innovation and improvement of physical and chemical treatments in WWTPs^2^,^5^,^6^.

In the past decades, substantial effort has been made to improve the contaminant removal efficiency of constructed wetlands (CWs). However, the removal of the excessive nutrients from polluted water remains a challenge in high-latitude areas or during winter in low- to middle-latitude areas with average temperatures of lower than 10 °C due to the significant relationship between temperature and the activity of both macrophytes and microorganisms^7^,^8^. Macrophyte species selection has been regarded as one technology to mitigate the decrease in wetland purification functions during winter^7^. For CWs with plants growing in a substrate, macrophytes are generally regarded as one of the most important components of CWs, but under certain conditions, the macrophyte functions are limited or even negligible^9^,^10^. However, for constructed floating wetlands (CFWs) with aquatic macrophytes growing in a floating matrix^11^, the conditions are different: the macrophytes (especially the roots) generally act as the main CFW component that play a dominant role in pollutant removal^12^. Numerous studies have been performed on species selection^9^,^13^,^14^,^15^,^16^. However, few studies have been focused on the purification capabilities of CFWs with cool-season aquatic macrophytes or on the importance of macrophytes in removing nutrients from polluted water in winter. The secondary effluent quantity from WWTPs is generally relatively constant between seasons, and consequently a relatively constant purification ability is required in the wetlands to treat the effluent, the possible improvement in removal efficiency in winter has special significance.

Moreover, little effort has been made to identify the contaminant removal pathway in CFWs during cold winters. The purification function of CFWs is known to be influenced by a number of processes, including plant absorption, litter decomposition, nitrification, denitrification and deposition^12^,^17^. For CWs with plants growing in a substrate, numerous studies have been conducted to quantify the purification function of aquatic macrophytes in the removal of pollutants and to understand the purification mechanism^8^,^18^. Ammonification followed by a series of nitrification and denitrification has been thought to be the primary pathway of nitrogen retention in CWs^18^,^19^. In CWs, the direct plant absorption and the purification function of aquatic macrophytes can be ignored under certain circumstances^8^,^9^. However, due to the absence of enough substrates and the limited absorption and microorganism activities, the purification pathway in CFWs is likely completely different^11^,^12^,^20^,^21^. Several studies have been conducted to identify the contaminant removal pathway in CFWs using warm-season species or study areas that experience only a few days with temperature below 10 °C in a given year^11^,^21^,^22^,^23^. However, little is known about the purification mechanism of CFWs in winter, such as whether the direct absorption by macrophytes or the activities of microbes play a more important role in the pollutant removal in winter?

Therefore, the primary aim of this study was to identify whether a cold-season aquatic macrophyte can facilitate the purification of a floating wetland in winter. If the facilitation by the cold-season macrophyte is positive, this plant type will likely be adopted widely by researchers and managers of CWs treating secondary effluent from WWTPs to improve contaminant removal efficiency in winter when purification ability greatly decreases. This study also sought to clarify the primary pathway of contaminant removal by CFWs, which is vital for the selection of management strategies. For example, if plant uptake is proved to be the main pathway in winter, plant growth rate should be used as the main parameter for species selection; if microbial activity is the dominant pathway in CFWs, the accumulation of biomass, especially root biomass, should be used as the indicative parameter to select for high root surface area for biofilm attachment^12^.

## Results

### Site measured parameters removal efficiency

**Figure 1:**
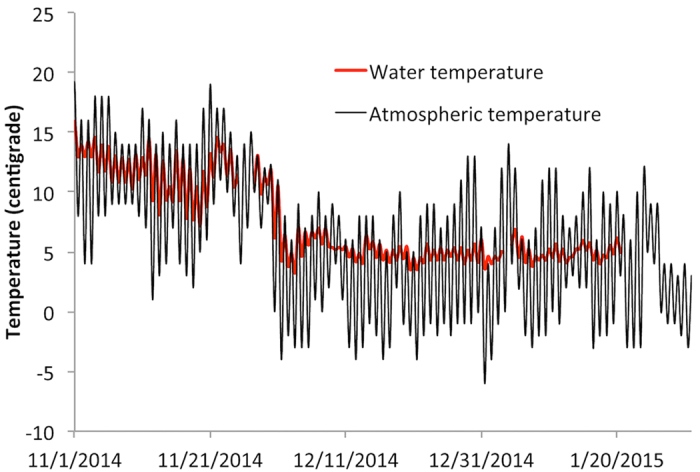
Continuous temperature series of the near-ground air and water in the floating wetland.

During the experimental period, the local atmospheric daily temperature fluctuated between −6.2 and 19.1 °C, with an average of 6.2 °C, and the water temperature (50 cm below the water surface) fluctuated between 3.3 and 15.9 °C, with an average of 7.52 °C (Fig. 1). The average air temperatures were 11.9, 9.45, 11.1, 3.25, 2.30, 3.85, 3.60 and 4.70 °C for the 1^st^, 2^nd^, 3^rd^, 4^th^, 5^th^, 6^th^ 7^th^ and 8^th^ batches, respectively; while the corresponding water temperatures were 12.7, 10.6, 11.6, 5.81, 4.89, 4.70, 4.82 and 4.99 °C, respectively. Based on air and water temperatures, the overall experimental period can be divided into two sub-periods: the first three batches (referred to as the late autumn batches in the following text) and latter five batches (the winter batches). Our experimental period featured both a warm autumn and a cold winter, suggesting the experimental period is appropriate for evaluating the purification ability of the cool-season aquatic macrophyte in FW in winter and comparing the performances between a cold winter and a warm autumn.

**Figure 2:**
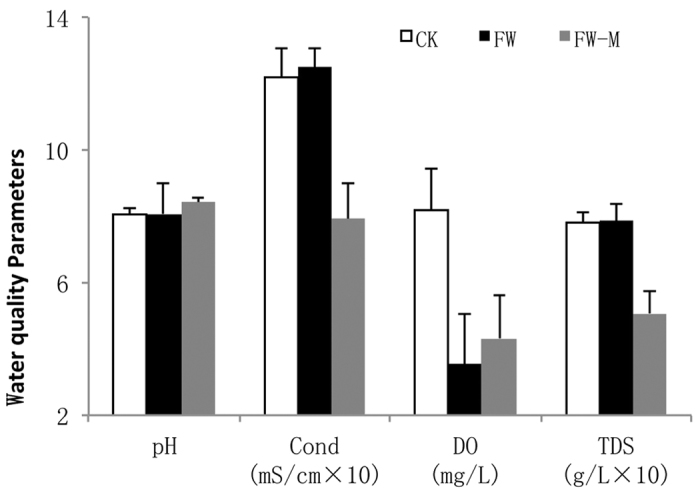
Field measurements of pH, conductivity, DO and TDS in the CK, FW and FW-M systems in winter (error bars represent standard deviation).

The average pH values were 8.0, 8.1 and 8.4 for the CK, FW and FW-M systems, respectively (Fig. 2), and the values exhibited no significant differences between treatments (*p* = 0.236). The FW-M treatment had a significantly lower conductivity value (0.79 mS/cm) than that of the CK (1.22 mS/cm, *p* = 0.003) and FW (1.25 mS/cm, *p* = 0.014) systems. Significant different TDS values were observed (*p* = 0.006), i.e., 0.78, 0.79 and 0.51 g/L for the CK, FW and FW-M systems, respectively. The CK system (8.21 mg/L) had a significantly higher DO than the FW (3.55 mg/L, *p* = 0.012) and FW-M (4.32 mg/L, *p* = 0.036) systems. These results suggest that *Oenanthe clecumbens* in floating wetlands can improve the water purification ability with respect to conductivity and TDS.

### Nutrition and COD removal efficiency

During the experimental period, all of the treatments had relatively low COD removal efficiencies, with average values of 8.13%, 14.59% and 18.53% for the CK, FW and FW-M systems, respectively (Table 1). For TN, NO_3_^−^-N, NH_4_^+^-N, TDN and TP, the average removal efficiency values were 26.48%, 36.18%, 39.30%, 26.23% and 24.64% in the FW-M system, 4.38%, 2.91%, 7.76%, 3.96% and 2.73% in the CK system, and 3.76%, 14.77%, 5.74%, 5.55% and 4.77% in the FW system, respectively. No significant differences were observed in the COD removal efficiency between treatments (*p* = 0.437), whereas FW-M generally had significant higher removal efficiencies for TN, NO_3_^−^-N, NH_4_^+^-N, TDN and TP than CK and FW (except for TN, TDN and TP in 8^th^ batch) (*p* values ranged from 0.002 to 0.016). For COD, TN, NO_3_^−^-N, NH_4_^+^-N, TDN and TP, the FE_fm_ values were 6.46%, −0.62%, 11.86%, −2.01%, 1.59% and 2.04%, and the FE_am_ values were 3.94%, 22.72%, 21.41%, 33.56%, 20.68% and 19.87%, respectively (Table S1). These results indicate that CFWs can efficiently remove N and P from secondary effluent, but the COD removal efficiency was relatively low. The presence of a cool-season aquatic macrophyte was the primary factor that improved the efficiency of N and P removal.

For the FW-M system, COD removal efficiency fluctuated slightly during the experimental period, whereas the TN, NO_3_^−^-N, NH_4_^+^-N, TDN and TP removal efficiencies generally decreased gradually with time, with the exception of the NH_4_^+^-N removal efficiency in 6^th^ batch, which was slightly higher than that in 4^th^ batch. FW-M removal efficiency decreased 14.57%, 16.47%, 16.11%, 10.03% and 24.06% in the winter batches over that in the autumn batches, for TN, NO_3_^−^-N, NH_4_^+^-N, TDN and TP, respectively (Table 1). For TN, NO_3_^−^-N, NH_4_^+^-N, TDN and TP in the winter batches, the FE_fw_ values were 16.99%, 28.50%, 25.24%, 15.57% and 18.68%, and the FE_am_ values were 17.37%, 18.25%, 25.86%, 15.23% and 15.24%, respectively (Table S1). These results suggest that although the purification ability of the CFWs decreased significantly in winter compared to late autumn (*p* = 0.003), the floating wetland still achieved N and P removal efficiencies of 20.22–35.28%. Moreover, the species *Oenanthe clecumbens* can improve the purification abilities of CFWs in winter, with FE_am_ values ranging from 15.23% to 25.86% for N and P.

### Plant growth dynamics

**Figure 3:**
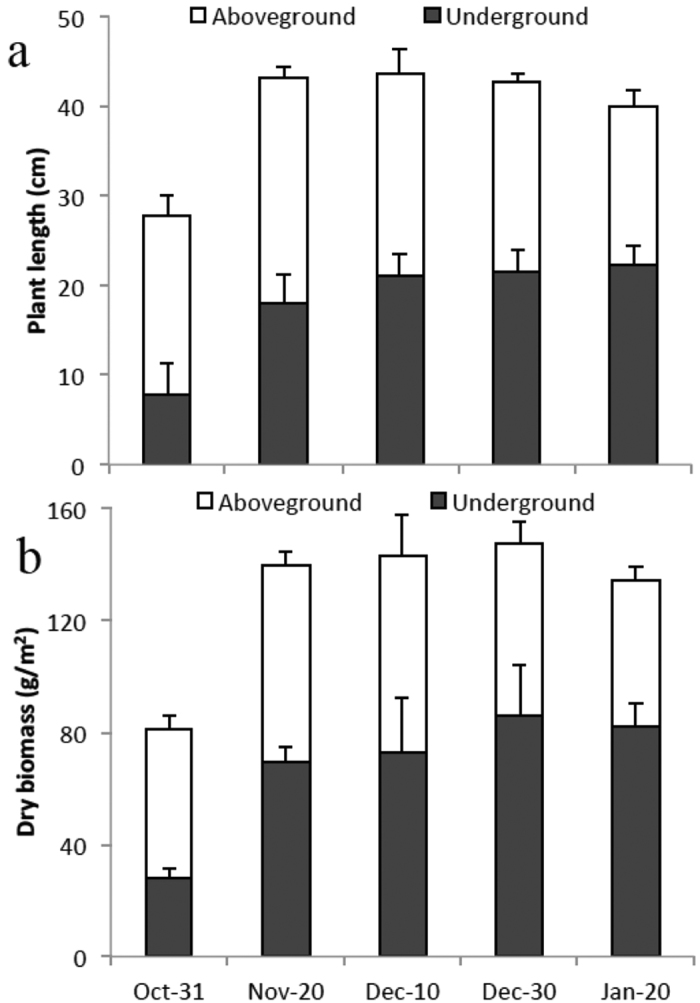
Growth characteristics of plant length (**a**) and dry biomass (**b**) of the *Oenanthe clecumbens* plants.

**Figure 4:**
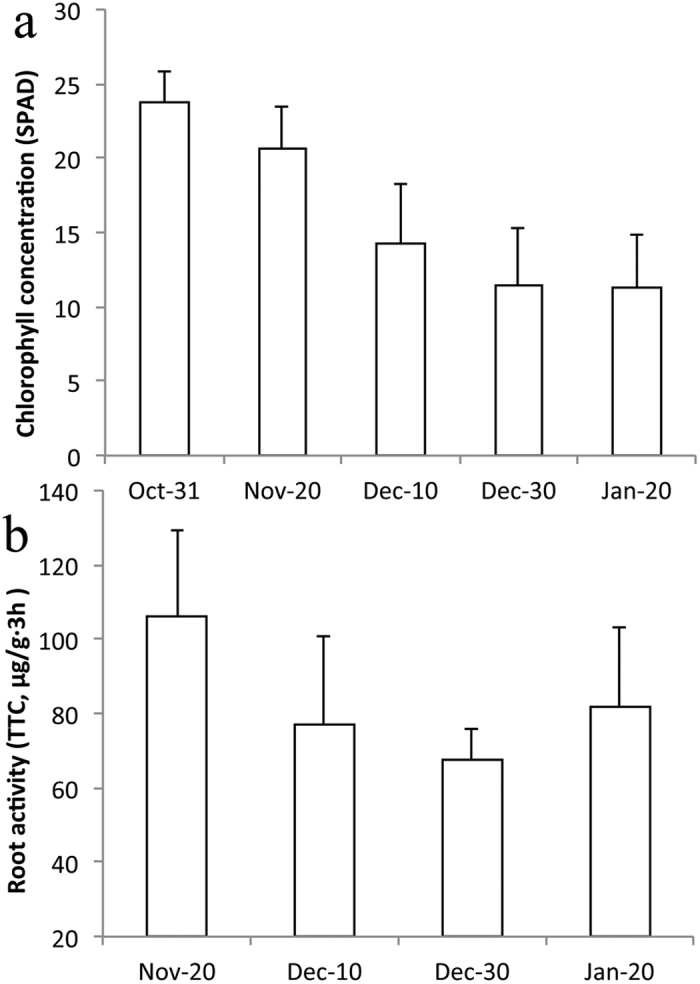
Growth characteristics of leaf chlorophyll concentration (**a**) and root activity (**b**) of the *Oenanthe clecumbens* plants.

In the FW-M system, the roots of *Oenanthe clecumbens* lengthened rapidly in the first two batches, from 7.8 cm on October 31 to 18.09 cm on November 20, and then lengthened slowly over the course of the next six batches (Fig. 3). The aboveground plant length also increased rapidly in the first two batches, from 20.02 cm to 25.15 cm, and then slowly decreased due to the withering of old leaves. The belowground and aboveground dry biomass behaved similarly to belowground and aboveground plant length, respectively. The leaf chlorophyll concentration gradually decreased over the entire experimental period, whereas root activity initially decreased gradually before December 30 and then increased slightly (Fig. 4). Therefore, although a portion of the leaves of the cold-season species *Oenanthe clecumbens* withered, the roots retained a relatively high degree of activity during a winter with average air and water temperatures of 3.63 and 5.04 °C, respectively.

### Nitrogen removal pathway

**Figure 5:**
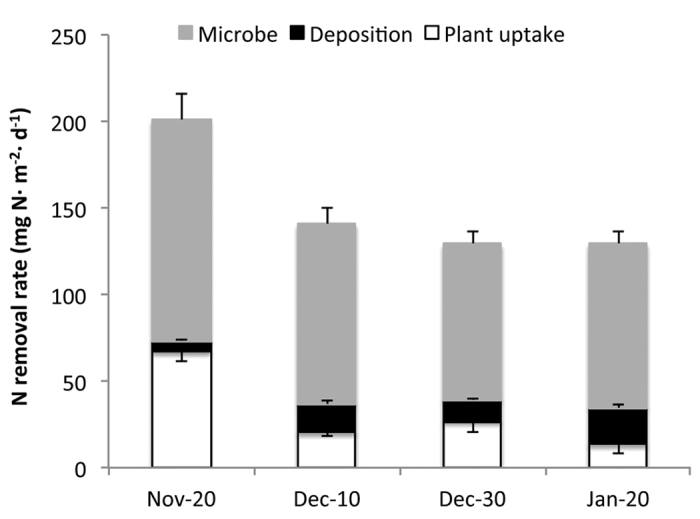
The contribution of microbial, deposition storage and plant uptake pathways to nitrogen removal in floating wetland systems.

For the FW-M system, the plant’s N uptake rate dramatically decreased from 67.13 mg N · m^−2^ · d^−1^ in the late autumn batch (2^nd^ batch) to an average of 20.09 mg N · m^−2^ · d^−1^ in the winter batches (4^th^, 6^th^ and 8^th^ batches). The plant’s N uptake rate decreased by an average of 70.07% between late autumn and winter batches (Fig. 5). Similarly, the proportion of total removed N represented by the plant uptake pathway decreased from 33.39% in the late autumn batch to an average of 15.01% in the winter batches. The N removal rate of the deposition storage pathway fluctuated between 6.25 and 21.26 mg N ·  m^−2^ · d^−1^ in the four studied batches, representing between 3.11% and 15.94% of the total N removal. The microbial N removal rate decreased from 127.66 mg N · m^−2^ · d^−1^ in the late autumn batch to an average of 96.34 mg N · m^−2^ · d^−1^ in the winter batches, a decrease of 24.53%. The microbial removal pathway proportion of the total N removal increased from 63.50% in the late autumn batch to 71.57% in the winter batches. The decreased N removal by the plant uptake and microbial pathways accounted for 69.38% and 46.19% of the decrease in total N removal, respectively, between late autumn and winter.

Based on our results, we have made the following observations. (1) For N removal in CFWs in winter, microbial removal was the most important pathway, followed by the plant uptake pathway, representing 71.57% and 15.01% of the total N removal, respectively. (2) The N removal rates of both the microbial and plant uptake pathways decreased in winter relative to late autumn, although the decrease in plant uptake (70.07%) was greatly larger than that of the microbial pathway (24.53%). (3) Although plant uptake was not the most important N removal pathway in both winter and late autumn, the decrease in the N uptake rate of the plants was the most important cause of the decrease (69.38%) in N removal efficiency in winter relative to late autumn.

## Discussion

Due to the great dependence on warm temperatures of the activity of microbes and macrophytes, the primary drivers of the removal of pollutants such as N and COD, constructing an effective wetland system in an area with cold winters (temperatures less than 10 °C) is challenging^7^,^24^,^25^. A dozen technologies to increase the effectiveness of wetlands in cold climates have been proposed and can be divided into three groups: internal improvements, winter operation and external incorporation^7^. The proper selection of plant species has been demonstrated to be an effective means to decrease heat loss from wetland systems^26^. Dozens of species have been recommended for constructed wetlands in Northern China, such as *Phragmites australis, Zizania latifolia* and *Typha orientalis*^7^. However, most of these proposed species become completely senescent by the end of November in most regions of China with minimum temperatures below zero, including our study area. Thus, these species are not actually suitable for the improvement of wetland purification in winter.

Consequently, the selection of new macrophyte species is necessary. Suitable species should retain at least some leaf activity and slow root growth throughout the winter. Partial leaf activity in winter can help resume rapid growth in the early spring when most of the previously proposed species, such as *Phragmites australis* and *Zizania latifolia*, are still in the germination period. This rapid spring growth would increase the recovery of the purification functions as soon as possible in the early spring. Additionally, the slow winter growth of the roots indicates that the roots maintain a relatively high degree of activity and purification abilities throughout the winter^12^. The species *Oenanthe clecumbens* studied in this paper probably meets these requirements because most of its leaves remain active and its roots continue to grow, thereby improving the contaminant removal rates in winter^12^ and resuming rapid growth in the early spring^27^. Our result, FE_am_ values ranging from 15.23% to 25.86% for N and P, confirmed this inference (We can infer the performance of warm-season plant CFW in winter according to the treatment of floating mats without plants because most warm-season plants in CFW can’t survive winter at our study area).

Macrophyte roots are believed to play a major role in the treatment processes in FTWs by providing surface area for biofilm attachment, secreting organic matter and promoting the flocculation of suspended matter^12^,^20^. The significant relationship between microbial activity and temperature resulted in the decrease in the N removal efficiency of microorganisms primarily in root biofilms in winter relative to autumn; thus, the N removed via the microbial pathway decreased in winter in this study^25^,^28^,^29^. The decrease in the secretion of organic matter by roots, which could be inferred from the decrease in the aboveground length and biomass of the plants in our study, may exacerbate the decrease in winter. However, due to (1) the increase in root surface area (inferred from the slight increase in root length and biomass in the winter batches relative to the late autumn batches), (2) the maintenance of root activity (demonstrated by both our results and other studies^27^ and (3) the possible presence of psychrotrophic bacteria^7^, N removal via the microbial pathway decreased only 24.53% in the winter batches compared to the late autumn batches. Moreover, another factor also contributes to the relatively high N and P removal efficiencies in winter in the FW-M system with overwintering macrophytes: a slight increase in the N removal efficiency of the deposition storage pathway is induced by the slight increase in *Oenanthe clecumbens* roots.

However, the condition of the plant uptake pathway is quite different. A plant’s nutrient uptake rate is determined by the plant’s growth rate and nutrient concentrations in the plant’s issues, both of which are influenced by many factors, including the plant species, growth period and nutrient concentrations in the influent^8^,^28^,^30^. Generally, the plant growth rate plays the dominant role. Few macrophyte species can maintain relatively high growth rates in environments below 5 °C; thus, improving nutrient removal rates is difficult to accomplish by increasing plant uptake in cold winter. Our result suggested that although plant uptake was not the dominant N removal pathway in general conditions, the decrease in plant uptake was the most important factor determining the decrease in wintertime N removal efficiency. Therefore, the selection of cold-season macrophytes can mitigate the decrease in CFW purification abilities in winter, mostly by maintaining relatively high N removal rates via the microbial and deposition storage pathways instead of the plant uptake pathway.

The N removal pathways also highlight the importance of the relatively large surface areas provided by roots, which can improve biofilm area, thereby increasing both the microbial and deposition storage N removal pathways^12^. Obtaining large surface areas of roots can be achieved via both species selection and proper wetland management. Species selection should aim to identify aquatic macrophyte species with large roots and high root/shoot ratios, and certain management practices, such as planting in early October or earlier, can result in larger plant biomasses and larger root surface areas before entering a cold winter.

We conclude that the presence of the cold-season plant *Oenanthe clecumbens* in CFWs significantly improved the removal of N and P during a winter with average air and water temperature of 3.63 and 5.04 °C, respectively. The N removal efficiency decreased significantly in winter compared to late autumn, primarily due to the decrease in the plant uptake pathway. Microbial removal and plant uptake accounted for 71.57% and 15.01% of the total removal of N in winter, respectively. The results suggest that the selection of cold-season plants is a reliable option for achieving high contaminant removal rates in CFWs and demonstrate the significance of roots with relatively high wintertime activity and biomass for further improving the FE_am_ values.

## Methods

### Site description and experimental design

The study was conducted in fiber-reinforced plastic (FRP) incubators (length = 2.1 m, width = 1.27 m, height = 0.66 m) at Nanjing University - Hongze Wetland Experimental Station (118°55′ E, 33°19′ N) in the Hongze Constructed Wetland for Secondary Wastewater (HZCWSW), Jiangsu Province, China. HZCWSW was established in 2012 to treat secondary wastewater from two local WWTPs and to improve the water quality by decreasing COD, NH_3_-N and TP from 60, 10 and 1 mg/L to 40, 2.0 and 0.4 mg/L, respectively, before the wastewater enters the Yellow Sea. The catchment has an average temperature of 15 °C, and an average annual precipitation of 1,080 mm. From 1986 to 2006, the average temperature in winter ranged between 2 and 5 °C.

Although several species with high nutrient uptake rates, such as *Iris louisiana*, *Zizania caduuciflora*, *Phragmites australis* and *Canna indica*, have been widely used in CFWs in the catchment, most are either deciduous plant species or are species with above-ground tissues that can survive winter if planted in soil but die in CFWs without soil. *Oenanthe clecumbens*, one of China’s winter specialty aquatic vegetables, has been used as a macrophyte in CFWs due to its low-temperature tolerance, high biomass and ability to be repeatedly harvested^27^,^31^.

To identify the purification function of cool-season aquatic macrophytes in winter, three types of systems were established: unplanted control without floating mats (CK), unplanted control with floating mats (FW) and planted floating mats (FW-M). Four replicates for treatments CK and FW and six replicates for treatment FW-M were constructed. The experimental CFW mesocosm study was carried out from October 18, 2014 to January 20, 2015. Seedlings of *Oenanthe clecumbens* were collected from a local nursery. Similarly sized seedlings (7–8 cm long) were selected, and their roots were cut to the same length (2 cm). The seedlings were then cleaned to remove the nutrient-rich soil from the nursery. The prepared seedlings were then transplanted into 9.0-cm-diameter hydroponic baskets (specially designed for HCFC500 floating mats from Hanchao Inc., http://www.gzhanchao.com). Four seedlings were planted in every basket. The baskets filled with plants were then fixed to the plastic floating mats (HCFC500, 50*50*6 cm, nine pots in each mat). Six prepared floating mats were then placed in each FRP incubator and fixed using iron wire.

Secondary wastewater was obtained from a neighboring wastewater treatment plant (WWTP), and the water’s characteristics are listed in Table 2. The wastewater filling and draining from the FRP were performed as a batch model, i.e., the wastewater was filled to a water depth of 50 cm at the start of each batch and then drained before the next batch. Before filling, each incubator was washed clean to remove the sediment (mostly combined by organic and inorganic particles and plant residues) formed at the previous batch. Each batch was 10 days (HRT = 10 d), and a total of 8 batches (80 d) were performed from October 31 in 2014 to January 20 in 2015. At the end of each batch, water level of each incubator was measured to calculate the changes of water volume.

### Water quality and plant status monitoring and analysis

The upper 20 cm of water in each incubator was sampled for water quality at the end of the 2^nd^, 4^th^, 6^th^ and 8^th^ batches. Several parameters were analyzed using a Hach DR/2400 spectrophotometer according to standard operating procedures: chemical oxygen demand (COD), total nitrogen (TN), NO_3_^−^-N, NH_4_^+^-N and TP. The total dissolved N (TDN) values were analyzed following standard methods (APHA, 1998)^32^. Particulate N was determined as the difference between TN and TDN. A multi-parameter water quality meter (HORIBA, U-52) was used to measure pH, conductivity, dissolved oxygen (DO) and total dissolved solid (TDS).

Immediately after water samples being taken, 9 baskets of plant were sampled for plant status measurements. Two baskets of plants were used to measure root activity via the triphenyl tetrazolium chloride (TTC) method^33^. The remaining 7 baskets of plants were used to measure the root and aboveground lengths, and the average growth speed was then calculated in cm·d^−1^. The 7 baskets of plants were divided into roots, leaves and stems, which were placed into brown paper bags, dried to a constant weight at 65 °C and weighed. The average growth speed was calculated for each FW-M treatment incubator in g·d^−1^·m^2^. The dried samples were then milled into a homogeneous powder, and the nitrogen content was measured via an elemental analyzer (Elemental Analyzer N A 2500). For each vegetation sampling, plants of one replicate of FW-M incubators were used to supplement the sampled plants in other replicates in order too maintain a constant vegetation density, and thus the replicates of FW-M changed from 6 at the beginning to 3 at the end of the experiment.

### Nitrogen removal pathway estimation

The pathway and ratio of nitrogen removal in the CFWs were estimated using the method suggested by Chen *et al.*^2^, which is only briefly described here. The nitrogen accumulation in the plants was calculated based on the aboveground and belowground biomasses multiplied by the respective N concentrations in the aboveground and belowground tissues. Plant uptake in a batch was calculated by subtracting the N content measured at the end of the previous batch from the accumulation rate in the end of the current batch. The nitrogen removed by the deposition was estimated as the difference between the particulate N in the influent and in the effluent. The N released from litter during the decomposition was ignored because most of the litter from *Oenanthe clecumbens* was above the floating mats and released little nitrogen to the water. The volatilization of ammonia is ignored due to the neutral pH of the influent. The microbial removal (mostly by denitrification) was estimated based on the difference between the overall removal rate and the deposition removal and plant uptake.

### Evaluation method

To evaluate the facilitation effect (FE) of floating mats and the aquatic macrophyte, three parameters were calculated:


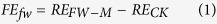



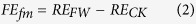



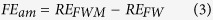


where FE_fw_, FE_fm_ and FE_am_ represent the FE due to the presence of floating mats with plants, floating mats without plants and only aquatic plants, respectively. The parameters RE_CK_, RE_FW_ and RE_FW-M_ represent the removal efficiencies of the CK, FW and FW-M systems, respectively.

Since most warm-season plants in CFW can’t survive winter in at our study area, the treatment of floating mats without plants was used to simulate the performance of CFW with warm-season plants in winter, and thus FE_am_ could be used to compare the performance of warm-season and cold-season plants in winter.

One-way and two-way analysis of variance (ANOVA) was conducted to test the significance of difference in purification ability among treatments or seasons using StatPlus^®: mac^ LE. 2009 (Version 5.8.2.0).

## Additional Information

**How to cite this article**: Zou, X. *et al.* Decreasing but still significant facilitation effect of cold-season macrophytes on wetlands purification function during cold winter. *Sci. Rep.* **6**, 27011; doi: 10.1038/srep27011 (2016).

## Supplementary Material

Supplementary Information

## Figures and Tables

**Table 1:** Removal efficiencies for COD, TN, NO_3_^−^-N, NH_4_^+^-N, TP and TDN in the constructed floating wetland systems (%).

Parameters	Treatments	Nov-20*	Dec-10*	Dec-30*	Jan-20*	Mean_w_#
COD	CK	4.28 a	9.32 a	10.16 a	8.75 a	9.41
	FW	11.69 a	12.67 a	17.74 a	16.26 a	15.55
	FW-M	17.73 a	22.33 a	14.78 a	19.28 a	18.80
TN	CK	0.01 b	7.94 b	4.11 b	5.49 b	5.84
	FW	−1.36 b	3.77 b	−3.04 b	15.66 ab	5.46
	FW-M	37.40 a	26.85 a	22.14 a	19.51 a	22.84
NO_3_-N	CK	0.98 b	3.69 b	1.23 b	5.75 b	3.56
	FW	17.65 b	20.08 ab	14.61 ab	6.75 b	13.81
	FW-M	48.53 a	39.21 a	38.27 a	18.70 a	32.06
NH_4_^+^-N	CK	0.93 b	9.34 b	16.20 b	4.57 b	10.04
	FW	−5.28 b	11.45 b	7.04 b	9.76 b	9.41
	FW-M	51.39 a	35.26 a	42.72 a	27.85 a	35.28
TP	CK	0.58 b	2.70 b	3.80 b	3.85 b	3.45
	FW	−1.61 b	7.60 b	8.29 b	4.81 b	6.90
	FW-M	32.16 a	31.81 a	23.81 a	10.78 a	22.13
TDN	CK	1.91 b	3.88 b	7.41 b	2.65 b	4.64
	FW	7.25 b	−2.52 b	4.63 b	12.85 ab	4.99
	FW-M	44.27 a	21.71 a	20.62 a	18.32 a	20.22

^*^Columns followed by the same letter are not significantly different at 95% confidence level.

^#^Means of the winter (i.e. the average of Dec-10, Dec-30 and Jan-20).

**Table 2:** Characteristics of the influent to the CFWs from a WWTP.

Parameters	COD	NH_4_^+^-N	NO_3_-N	TN	TP	TDN
Concentration (mg/L)^a^	38.65 ± 4.63	7.33 ± 1.43	3.12 ± 1.32	13.98 ± 1.73	6.43 ± 1.82	13.55 ± 1.12

^a^Values are given as the mean ± standard deviation (n = 16).
