# Supplementary material for: Decreasing but still significant facilitation effect of cold-season macrophytes on wetlands purification function during cold winter
Source: Sci Rep. 2016 Jun 1;6:27011. doi: 10.1038/srep27011 (PMC5378900; doi:10.1038/srep27011)
Supplement: Supplementary Information [file srep27011-s1.doc]

Decreasing but still significant facilitation effect of cold-season macrophytes on wetlands purification function during cold winter

Xiangxv Zou, Hui Zhang, Jie Zuo, Penghe Wang, Dehua Zhao[[1]](#footnote-2), Shuqing An

School of Life Sciences, Nanjing University, Nanjing 210046, PR China

Table S1 Average values of facilitation effect of floating mats and the aquatic macrophyte for all batches, autumn batches and winter batches (%).

|  | All batches | | |  | Autumn batches | | |  | Winter batches | | |
| --- | --- | --- | --- | --- | --- | --- | --- | --- | --- | --- | --- |
|  | FE_fw_ | FE_fm_ | FE_am_ |  | FE_fw_ | FE_fm_ | FE_am_ |  | FE_fw_ | FE_fm_ | FE_am_ |
| COD | 10.40 | 6.46 | 3.94 |  | 13.45 | 7.41 | 6.04 |  | 9.39 | 6.15 | 3.24 |
| TN | 22.09 | -0.62 | 22.72 |  | 37.40 | -1.36 | 38.76 |  | 16.99 | -0.38 | 17.37 |
| NO_3_^-^-N | 33.26 | 11.86 | 21.41 |  | 47.55 | 16.67 | 30.88 |  | 28.50 | 10.25 | 18.25 |
| NH_4_^+^-N | 31.55 | -2.02 | 33.56 |  | 50.46 | -6.20 | 56.67 |  | 25.24 | -0.62 | 25.86 |
| TP | 21.91 | 2.04 | 19.87 |  | 31.58 | -2.19 | 33.77 |  | 18.68 | 3.45 | 15.24 |
| TDN | 22.27 | 1.59 | 20.68 |  | 42.37 | 5.34 | 37.02 |  | 15.57 | 0.34 | 15.23 |

1. Corresponding author. Tel./fax: +86 025 89684560.

   E-mail address: [dhzhao@nju.edu.cn](mailto:dhzhao@nju.edu.cn) (D. Zhao). [↑](#footnote-ref-2)
